# Cysteine improves boar sperm quality via glutathione biosynthesis during the liquid storage

**DOI:** 10.5713/ab.21.0151

**Published:** 2021-08-22

**Authors:** Zhendong Zhu, Yao Zeng, Wenxian Zeng

**Affiliations:** 1 College of Animal Science and Technology, Qingdao Agricultural University, Qingdao 266109, China; 2 Key Laboratory of Animal Genetics, Breeding and Reproduction of Shaanxi Province, College of Animal Science and Technology, Northwest A&F University, Shaanxi 712100, China

**Keywords:** Boar Sperm, Cysteine, Glutathione Synthesis, Reactive Oxygen Species Stress

## Abstract

**Objective:**

Sperm is particularly susceptible to reactive oxygen species (ROS) stress. Glutathione (GSH) is an endogenous antioxidant that regulates sperm redox homeostasis. However, it is not clear whether boar sperm could utilize cysteine for synthesis GSH to protect sperm quality from ROS damage. Therefore, the present study was undertaken to elucidate the mechanism of how cysteine is involved in protecting boar sperm quality during liquid storage.

**Methods:**

Sperm motility, membrane integrity, lipid peroxidation, 4-hydroxyIlonenal (4-HNE) modifications, mitochondrial membrane potential, as well as the levels of ROS, GSH, and, ATP were evaluated. Moreover, the enzymes (GCLC: glutamate cysteine ligase; GSS: glutathione synthetase) that are involved in glutathione synthesis from cysteine precursor were detected by western blotting.

**Results:**

Compared to the control, addition of 1.25 mM cysteine to the liquid storage significantly increased boar sperm progressive motility, straight-line velocity, curvilinear velocity, beat-cross frequency, membrane integrity, mitochondrial membrane potential, ATP level, acrosome integrity, activities of superoxide dismutase and catalase, and GSH level, while reducing the ROS level, lipid peroxidation and 4-HNE modifications. It was also observed that the GCLC and GSS were expressed in boar sperm. Interestingly, when we used menadione to induce sperm with ROS stress, the menadione associated damages were observed to be reduced by the cysteine supplementation. Moreover, compared to the cysteine treatment, the γ-glutamylcysteine synthetase (γ-GCS) activity, GSH level, mitochondrial membrane potential, ATP level, membrane integrity and progressive motility in boar sperm were decreased by supplementing with an inhibitor of GSH synthesis, buthionine sulfoximine.

**Conclusion:**

These data suggest that boar sperm could biosynthesize the GSH from cysteine *in vitro*. Therefore, during storage, addition of cysteine improves boar sperm quality via enhancing the GSH synthesis to resist ROS stress.

## INTRODUCTION

Reactive oxygen species (ROS) is a byproduct of oxidative phosphorylation in mitochondria [1]. The excessive ROS targets the electron transfer chain proteins and mitochondrial transcription factors including specific RNA polymerase and mitochondrial transcription factor-A, and in turn, disrupt the ATP generation and gene expression systems in mitochondria [2]. Supplementation of the exogenous mitochondria-target antioxidants improves boar sperm linear motility by protecting the gene transcription system and ATP generation system [2,3]. Thus, reducing the ROS stress is suggested to an essential strategy for maintaining boar sperm quality.

Mammalian sperm is a kind of redox-regulated cell that balances the redox homeostasis with its endogenous antioxidant defense systems [4,5]. Particularly, glutathione (GSH) is present in mammalian sperm [6–8] and is one of the most essential intracellular factors for regulating the redox homeostasis in sperm [8,9]. Additionally, the GSH level of sperm is decreased under the oxidative stress induced by cryopreservation [8,10,11] and incubation [12] processes. Addition of the GSH to the freezing medium or liquid extender improved sperm quality via scavenging the ROS in boars, bovines, humans, and rams [8,10,12,13]. Moreover, Takeo and Nakagata [14] reported that supplementation of GSH to mouse sperm increased the fertilization ratio as well *in vitro*.

The GSH is generated from glutamate, cysteine, and glycine via two enzymatic steps in somatic cells; first, γ-glutamylcysteine is synthesized from cysteine and glutamate by γ-glutamylcysteine synthase (GCLC). The second step is catalyzed by glutathione synthase (GSS) and comprises covalent linkage of glycine to γ-glutamylcysteine [15]. Importantly, the availability of cysteine is the rate-limiting amino acid for GSH synthesis in somatic cells [15,16]. Addition of cysteine to the freezing medium significantly enhanced the GSH level in rabbit sperm [7]. Moreover, previous studies reported that the addition of cysteine to the extenders improved sperm quality in chicken [17], caprine [18], stallion [19], and boar [20] during liquid storage. In stallion sperm, Ortega-Ferrusola et al [21] reported that addition of cysteine to the medium increased the sperm GSH level during *in vitro* incubation, which indicated that stallion sperm could utilize the exogenous cysteine for the biosynthesis of GSH in responding to the ROS stress. In addition, Lee et al [20] showed that addition of S-allyl-L-cysteine to medium could protect boar sperm motility, plasma membrane integrity and mitochondrial activity. However, the mechanism of how cysteine protects boar sperm is unclear. Therefore, our hypothesis was that boar sperm might use the cysteine for GSH synthesis and thus maintain cell structure and function during liquid storage. Hence, the present study was aimed to elucidate how the cysteine protects boar sperm quality during liquid storage.

## MATERIALS AND METHODS

### Chemicals and extenders

Routine chemicals and reagents were purchased from Sigma-Aldrich, China unless otherwise specified. Modena solution was used as the diluted extender in this study. The Modena solution was prepared according to our previous study [22].

### Animals, semen collection and processing

All animal treatments and experimental procedures were approved by the Northwest A&F University Institutional Animal Care and Use Committee (H18-11). Seven fertile and mature Duroc boars were housed individually, maintained under natural daylight, fed basal diets, and provided free access to water. The sperm-rich fraction was collected weekly from each boar with gloved-hand technique and filtered using a double gauze. Samples from the collected semen were evaluated for motility and morphology under light microscopy. Only the ejaculates containing sperm with more than 80% motile and 80% normal morphology were used in this study. The ejaculated semen was pooled to avoid individual differences. The mixed samples were divided into nine groups and diluted with Modena solution at a concentration of 5.0×10^7^ sperm/mL. First, five groups of diluted semen were treated with different concentrations of cysteine (0, 0.625, 1.25, 2.5, and 5 mM), then split into 80 mL semen doses and stored at 17°C in a cool incubator (BC-43KT1; Hisense Co., Qingdao, China) before evaluation in experiment I. The other groups of diluted semen were incubated with menadione, cysteine or buthionine sulfoximine (BSO) in experiment II.

### Evaluation of sperm motility by computer-assisted sperm analysis

According to our previous study [23], sperm motility was assessed with computer-assisted sperm analysis (CASA) system (Integrated Semen Analysis System; Hview, Fuzhou, China). First, 5 μL of semen sample was added to the prewarmed analyzer’s Makler chamber, and three fields were randomly selected for CASA to assess sperm motility in each treatment. Total motility: Percentage of motile sperm moving with a path velocity >12 μm/s. Progressive motility: Percentage of motile sperm moving with path velocity 45 μm/s and in a straight line for over 80% of the time.

### Sperm membrane integrity and acrosome integrity

According to our previous study, sperm membrane integrity and acrosome integrity were measured with LIVE/DEAD Sperm Viability Kit and fluorescein isothiocyanate-peanut agglutinin, respectively. The stained sperm was monitored and photographed with an epifluorescence microscope according to Zhu et al [23].

### Mitochondrial membrane potential

Sperm mitochondrial activity was analyzed with the JC-Mitochondrial Membrane Potential Detection Kit (Beyotime Institue of Biotechnology, Shanghai, China) according to our previous study [3]. Briefly, sperm samples were stained with 1x JC-1 working solution for 30 min in dark. Monomer and aggregates are the two types of JC-1 stained mitochondrial plasma. The monomer emits green fluoresce while the aggregates emit red fluoresce. A monochromator microplate reader (Safire II, Tecan, Switzerland) was used to detect the fluorescence intensity of monomer (λem 525 nm) and aggregates (λem 590 nm). The sperm mitochondrial membrane potential was calculated as the fluorescence ratio of red to green. Analyses were performed in triplicate (n = 3).

### Measurement of sperm reactive oxygen species level

Sperm F level was measured with Reactive Oxygen Species Assay Kit (Beyotime Institute of Biotechnology, Shanghai, China) according to our previous study [6]. Sperm samples were incubated with 10 μM 2′,7′-Dichlorofluorescin diacetate (DCFH-DA) at 37°C for 30 min. The stained sperm was washed three times to remove the unbound probe and analyzed with a microplate reader (Synergy HT, BioTek, Winooski, VT, USA) at 485 nm excitation and 535 nm emission. Analyses were performed in triplicate (n = 3).

### Measurement of sperm ATP level

Sperm ATP level was measured with an ATP Assay Kit (Beyotime Institute of Biotechnology, China) according to the previous study [23]. Briefly, the samples were mixed with the assay buffer and substrates, and then measured the luminescence with a luminometer (Thermo Scientific, Palm Beach, FL, USA). Analyses were performed in triplicate (n = 3).

### Detection of γ-GCS activity

A γ-glutamylcysteine synthetase (γ-GCS) Assay Kit (Nanjing Jiancheng Bioengineering Institute, Nanjing, China) was used to detect the sperm γ-GCS activity. According to Zhu et al [6], sperm samples were lysed by ultrasonication (20 kHz, 750 W, operating at 40% power, 5 cycles of 3 son and 5 s off) and centrifuged at 12,000 g for 10 min at 4°C. Following the instructions of the kit, the supernatants were used to measure γ-GCS activity using a microplate reader with the absorbance at 636 nm. γ-GCS activity is expressed as units per mg protein. Protein concentrations were determined using Bradford’s method with bovine serum albumin (BSA) as the standard. Analyses were performed in triplicate (n = 3).

### Measurement of total GSH

Total GSH level was measured with a Glutathione Quantification Kit (Beyotime Institute of Biotechnology, China) according to Zhu et al [7]. Briefly, the sperm samples were lysed by three cycles of rapid cooling in liquid nitrogen and thawing at 37°C, centrifuged at 10,000 g for 10 min. After that, the supernatants were transferred to a 96-well plate to measure sperm total GSH level following the manufacturer’s introductions. Analyses were performed in triplicate (n = 3).

### Evaluation of boar sperm superoxide dismutase and catalase activities

According to Zhu et al [3], activities of sperm superoxide dismutase (SOD) and catalase were analyzed with total SOD assay kit and catalase assay kit respectively (Beyotime Institute of Biotechnology, China). Sperm samples were lysed ultrasonically (20 kHz, 750 W, operating at 40%, on 3 s, off 5 s, 5 cycles) on ice and centrifuged at 12,000 g for 10 min at 4°C to collect the supernatants. Then, the supernatants were used to analyze the activities of SOD and catalase according to the manufacturer’s introduction. Analyses were performed in triplicate (n = 3).

### Immunofluorescence

Sperm were spread onto glass slides and fixed with methanol for 10 min. Then, the samples were permeabilized with 0.5% Triton X-100 in phosphate-buffered saline (PBS). Nonspecific binding was blocked with PBS containing 10% BSA (w/v) (Life Technologies, Grand Island, NY, USA) for 30 min at 25°C. The sperm were incubated with primary anti-4HNE (ab48506; Abcam, Cambridge, UK) overnight. Next day, the sperm were washed three times in PBS and incubated with goat anti-mouse (1:100, sc-516141; Santa Cruz Biotechnology, Paso robles, CA, USA) antibody for immunofluorescence labeling. Subsequently, digital images were captured using a fluorescence microscope (80i; Nikon, Tokyo, Japan). The negative controls were treated without the primary antibodies.

### Western blotting

According to our previous studies [22,24], the proteins were separated by 12.5% sodium dodecyl sulfate-polyacrylamide gel electrophoresis and transferred to polyvinylidene difluoride membranes (GE Bioscience, Newark, NJ, USA). Non-specific binding sites were blocked by incubation in Tris-buffered saline (TBS) containing 0.1% (v/v) Tween-20 and 5% (w/v) bovine serum albumin (Life Technologies, USA). The membranes were immunoblotted with anti-GCLC (ARP54577-P050, Aviva Systems Biology, San Diego, CA, USA), anti-GSS (sc-166882, Santa Cruz Biotechnology, USA), 4 hydroxynonenal (4-HNE) and anti-α-tubulin (2148; Cell Signaling Technology, lnc., Danvers, MA, USA) antibodies diluted with 5% BSA in TBS-Tween (1:1,000 dilution) overnight at 4°C. Followed by incubation with the HRP-conjugated secondary antibodies (goat anti-rabbit antibody [14708S; Cell Signaling Technology, lnc., USA] for GCLC and α-tubulin; goat anti-mouse [ab205719; Abcam, USA] for GSS and 4-HNE; 1:5,000 dilution). After washing in TBST, enhanced chemiluminescence (ECL) detection was performed using the ECL system according to the manufacturer’s specifications (GE Bioscience, USA), and appropriate exposure of blots to Fuji x-ray film (Fujifilm, Tokyo, Japan). Band intensities were analyzed using a Gel-Pro Analyzer (Media Cybernetics0, Rockville, MD, USA).

### Experiment design

Experiment 1 was aimed to evaluate whether the exogenous cysteine was beneficial to boar sperm during liquid storage. Boar semen were diluted with Modena solution supplemented with different concentrations of cysteine (0, 0.625, 1.25, 2.5, 5 mM) during liquid storage. Sperm motility patterns, membrane integrity, ROS level, lipid peroxidation, 4-HNE modifications, GSH level, activities of SOD and catalase, mitochondrial membrane potential, ATP level and acrosome integrity were analyzed during the storage.

Experiment 2 was to investigate whether boar sperm metabolized cysteine for the biosynthesis of GSH to improve sperm quality via reducing the ROS damage. Menadione was used to induce a model with ROS stress in boar sperm, and the GCLC selective inhibitor, BSO was used to block GSH synthesis in this study. The treatments were as follows: i) a control group, incubated without menadione, cysteine or BSO; ii) a group incubated with 30 μM menadione; iii) a group incubated with 30 μM menadione and 1.25 mM cysteine; iv) a group incubated with 30 μM menadione, 1.25 mM cysteine and 100 μM BSO. Sperm were incubated with those four treatments for 3 h. The GCLC and GSS enzymes in boar sperm was detected by western blotting, and sperm ROS level, GSH level, γ-GCS activity, mitochondrial membrane potential, ATP level, membrane integrity and progressive motility were also analyzed.

### Statistical analysis

Data from three replicates were compared using either Student’s t-test or one-way analysis of variance followed by Tukey’s post hoc test (Statview; Abacus Concepts, Inc., Berkeley, CA, USA). All the values are presented as the mean±standard deviation. Treatments were considered statistically different from one another at p<0.05.

## RESULTS

### Effects of cysteine on sperm motility patterns and membrane integrity during the liquid storage

**Figure 1:**
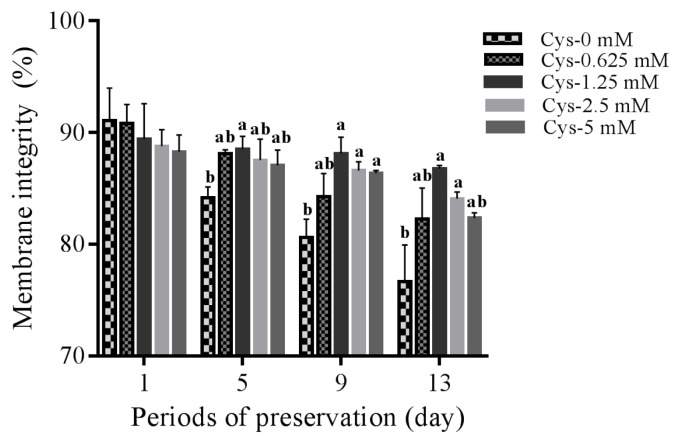
Dynamic changes in the sperm membrane integrity by addition of different concentrations of cysteine to the diluted medium after 13 days’ preservation. Values are specified as mean±standard deviation (SD). ^a,b^ Columns with different lowercase letters differ significantly (p<0.05).

As shown in Table 1, addition of cysteine did not improve the total motility during 9 days of preservation. But addition of 1.25 mM cysteine improved sperm total motility at 13-day point of preservation (Table 1). In terms of sperm progressive motility, addition of cysteine (from 1.25 to 5 mM) significantly improved it at 5-day, 9-day, and 13-day points of preservation (Table 2). Interestingly, it was also observed that addition of 1.25 mM cysteine significantly increased the sperm straight-line velocity, curvilinear velocity, and beat-cross frequency, while the other treatments did not improve them when compared to the control group (Tables 3–5). Moreover, the values of sperm membrane integrity were also significantly increased by addition of cysteine from 1.25 and 2.5 mM at 5-day, 9-day, and 13-day points of storage, while there is no change at the 1-day of storage. Interestingly, the 1.25 mM dose of cysteine showed the highest value among all treatments (Figure 1).

### Effects of cysteine on sperm total GSH level, activities of SOD and catalase, ROS level and lipid peroxidation during the liquid storage

**Figure 2:**
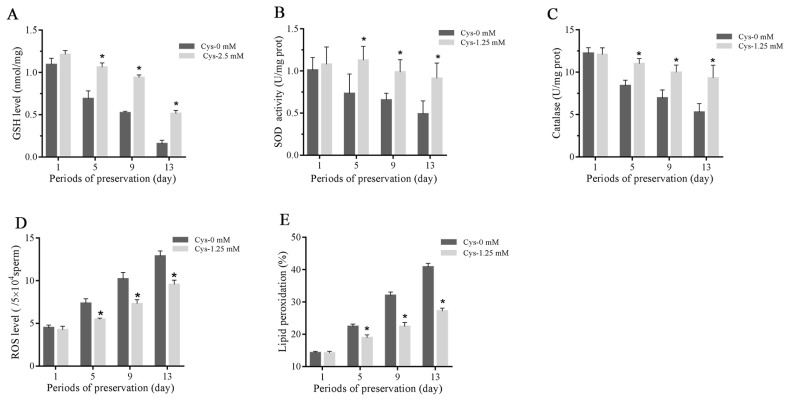
Effects of cysteine on sperm GSH level (A), activities of SOD (B) and catalase (C), ROS level (E) and lipid peroxidation (F) after 13 days’ preservation. Values are specified as mean±standard deviation (SD). GSH, glutathione; SOD, superoxide dismutase; ROS, reactive oxygen species; Cys, cysteine. * Denotes significant differences compared with the control (p<0.05).

As shown in Figure 2A–2C, the sperm total GSH level, activities of SOD and catalase were decreased during the liquid storage, and addition of 1.25 mM cysteine significantly increased the GSH level and activities of SOD and catalase compared to the control. The ROS level was increased during the liquid storage, while it was lower in the cysteine treatment than in the control (Figure 2D). Moreover, the result of sperm lipid peroxidation is similar to the ROS level in which addition of cysteine significantly decreased the value of lipid peroxidation compared to the control (Figure 2E).

### Effects of cysteine on sperm 4-hydroxynonenal modifications

**Figure 3:**
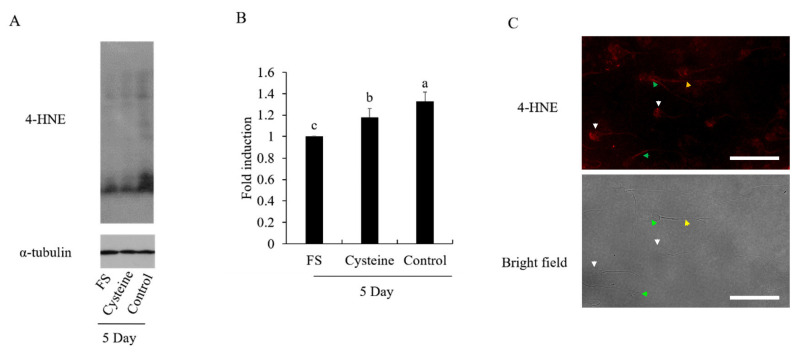
(A–B) Western blotting analysis of the effect of cysteine on 4-HNE modifications in boar sperm after 5-days’ preservation. Western blotting image is showing the expression of 4-HNE modifications in boar sperm (A). (B) Quantitative expression of the 4-HNE over α-tubulin generated from western blotting (A). Immunofluorescent localization of 4-HNE in boar sperm (C). Data are the mean±standard deviation (SD). 4-HNE, 4-hydroxyIlonenal. ^a–c^ Columns with different lowercase letters differ significantly (p<0.05). Bars = 30 μm.

To further investigate how the cysteine protects boar sperm during the liquid storage, we measured the 4-HNE modifications in sperm. It was observed that the 4-HNE level was significantly increased after 5 days of storage, when compared to the fresh sperm (Figures 3A–B). In addition, addition of cysteine to the diluted medium decreased the sperm 4-HNE modifications compared to the control (Figures 3A–B). Interestingly, it was observed that the 4-HNE was expressed in the head, midpiece, and tail of the sperm, and the signals in the head and midpiece were much stronger that tail (Figure 3C).

### Effects of cysteine on sperm mitochondrial membrane potential, ATP level and acrosome integrity during the storage

**Figure 4:**
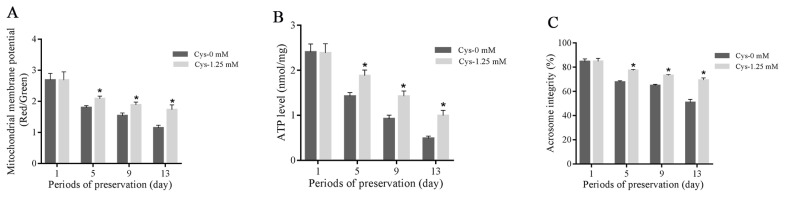
Effects of cysteine on sperm mitochondrial membrane potential (A), ATP level (B) and acrosome integrity (C) after 13 days’ preservation. Values are specified as mean± standard deviation (SD). Cys, cysteine. * Denotes significant differences compared with the control (p<0.05).

As shown in Figure 4A–C, sperm mitochondrial membrane potential, ATP level and acrosome integrity were decreased with the time extension during the liquid storage, and the addition of cysteine significantly increased those parameters when compared to the control at 5-day, 9-day, and 13-day points of storage. But no significant change was observed at 1-day’s storage (Figures 4A–C).

### Expression of GSS and GCLC enzymes in boar sperm

**Figure 5:**
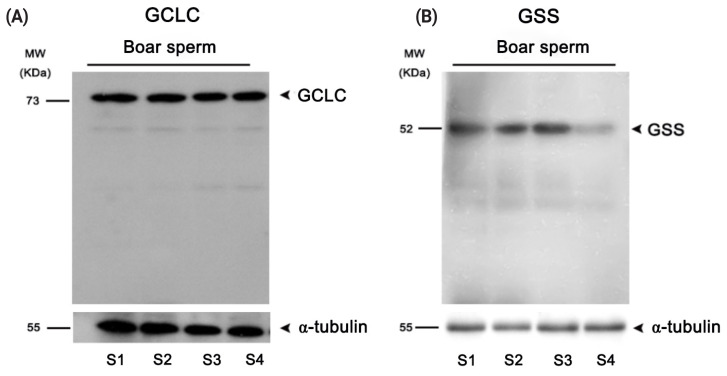
Western blotting detection of (A) the GCLC and (B) GSS enzymes in boar sperm. GCLC, glutamate cysteine ligase; GSS, glutathione synthetase; S1–4, sample1–4.

Western blotting analysis was performed to determine whether the GSS and GCLC enzymes were expressed in boar sperm. As showed in Figures 5A–B, those two key enzymes involved in GSH synthesis were expressed in the boar sperm.

### Effects of cysteine on sperm GSH biosynthesis and sperm quality under an oxidative stress model induced by menadione

**Figure 6:**
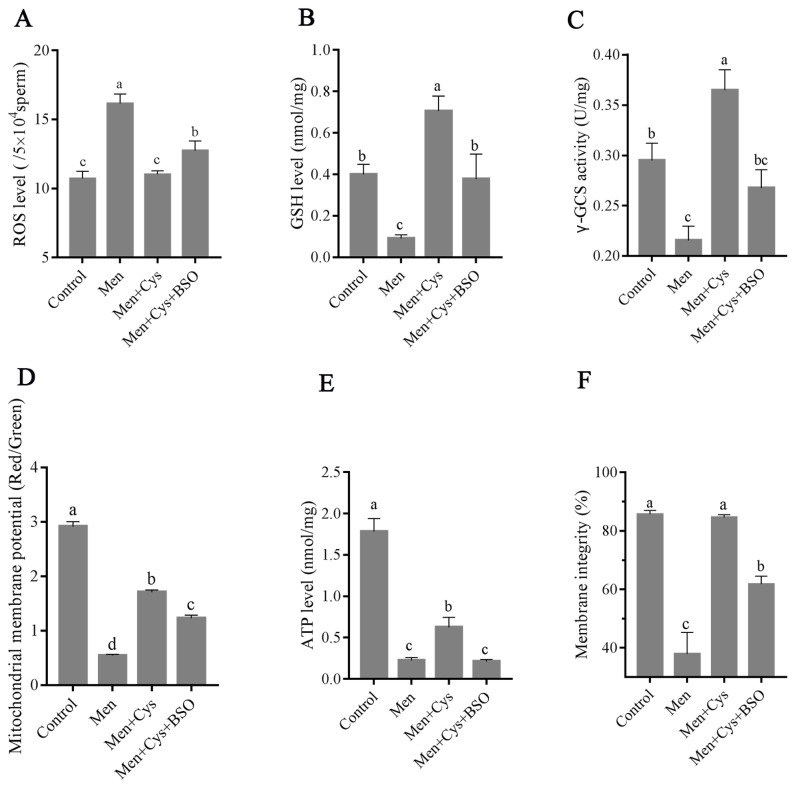
Effect of cysteine on the GSH level and the ROS damage induced by menadione *in vitro* condition. ROS level (A), GSH level (B), γ-GCS activity (C), mitochondrial membrane potential (D), ATP level (E) and membrane integrity (F). Data are the mean±standard deviation (SD). Cys, cysteine; Men, menadione; BSO, buthionine sulfoximine; γ-GCS, γ-glutamylcysteine synthetase. ^a–c^ Columns with different lowercase letters differ significantly (p<0.05).

We incubated sperm with 30 μM menadione for 3 h to induce oxidative stress *in vitro*. As shown in Figure 6A, the ROS level in the menadione treatment was much higher that of control, and addition of cysteine decreased the sperm ROS level. Interestingly, the positive effect of cysteine on ROS level was counteracted in presence of 100 μM BSO which is an inhibitor of GSH, (Figure 6A). Moreover, it was observed that the menadione treatment significantly decreased GSH level compared to the control (Figure 6B). Interestingly, supplementation of cysteine to the menadione treatment increased the GSH level, however, the positive effect was counteracted by addition of BSO to the incubation medium (Figure 6B). γ-GCS enzyme is a key protein involved in GSH biosynthesis from cysteine. The γ-GCS activity is one of the limiting factors for GSH generation. Comparing to the control, sperm treated with 30 μM menadione decreased γ-GCS activity, whereas addition of cysteine significantly increased it (Figure 6C). But the γ-GCS activity was decreased in presence of BSO, compared to the cysteine treatment (Figure 6C).

In terms of mitochondrial membrane potential, ATP level and membrane integrity, it was observed that those parameters were significantly increased with the addition of cysteine to the incubation medium compared to the menadione treatment, but those positive effects were counteracted by addition of BSO (Figures 6D–F).

**Figure 7:**
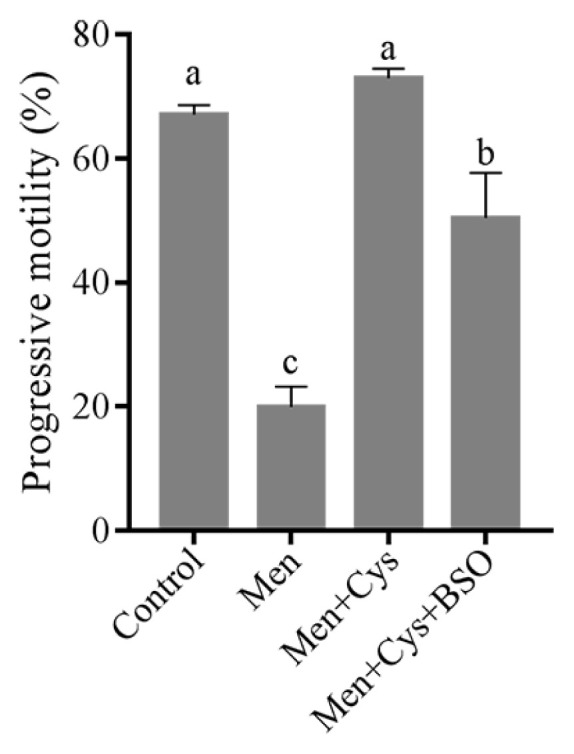
Effect of cysteine on sperm progressive motility in the menadione-induced ROS stress model. Data are the mean±standard deviation (SD). ^a–c^ Columns with different lowercase letters differ significantly (p<0.05).

In addition, when we analyzed the sperm progressive motility, we found that the menadione significantly decreased sperm progressive motility, while addition of cysteine could maintain it when compared to the control. Interestingly, the positive effect of cysteine on sperm progressive motility was counteracted by addition of BSO to the medium (Figure 7).

## DISCUSSION

Sperm is a kind of redox-regulated cell that balances the redox homeostasis with its endogenous antioxidant defense systems under physiological conditions [4,5]. GSH, a well-known non-enzyme antioxidant, is present not only in somatic cells but also in sperm [6–8]. GSH was one of the most essential intracellular factors for regulating redox homeostasis in sperm [8,9]. Addition of GSH to the freezing medium or liquid extender improved sperm quality via scavenging the ROS in boars, bovines, humans, and rams [8,10,12,13]. In the present study, we found that supplementation of cysteine led to an increase of the sperm GSH level, a reduction of ROS level, lipid peroxidation and 4-HNE modifications, and an increase of sperm progressive motility, mitochondrial membrane potential as well as ATP production. These data were consistent with the previous studies [8,10,12,13], indicating that the sperm GSH level is important to minimize the inevitable risk of oxidative stress.

In somatic cells, the glycine, cysteine and glutamate amino acids are the substrates for GSH *de novo* biosynthesis [16]. Cysteine is the limiting substrate for GSH biosynthesis as its level is very low compared to other two substrates. The cells usually uptake the exogenous cysteine or metabolize the methionine to generate cysteine for GSH production [15,16, 25]. In previous studies, we found that addition of cysteine and glutamine to the freezing medium could significantly increase the GSH level in rabbit sperm [6,7]. Moreover, Ortega-Ferrusola et al [21] found that the GCLC and GSS enzymes were expressed in stallion sperm, and the sperm could synthesis GSH using the exogenous cysteine [21]. In the present study, we observed that boar sperm also expressed the GCLC and GSS enzymes, and addition of cysteine to the extender increased sperm GSH level during the liquid storage, suggesting that boar sperm could synthesize GSH *in vitro* in response to ROS stress. Further evidence of GSH synthesis in boar sperm was confirmed by incubating boar sperm with BSO (a specific inhibitor of GCLC) in present of cysteine. This is the first time that it has been shown that boar sperm can biosynthesize GSH to reduce ROS stress *in vitro*.

Mammalian sperm are sensitive to ROS stress [2,26–28]. Though there are cellular enzymatic and nonenzymatic antioxidant defenses systems to keep the ROS balance [29] in somatic cells, the antioxidant defense systems in sperm is very limited as most of the cytoplasm is despoiled during spermiogenesis [30,31]. The excessive ROS leads to damage of sperm structure and function [32–35]. In the previous study, we found that ROS not only damaged the mitochondrial transcription factors including mitochondrial transcription factor-A and RNA polymerase, but also impaired the mitochondrial DNA integrity, nicotinamide adenine dinucleotide phosphate (NADPH) dehydrogenase subunits 1 and NADPH dehydrogenase subunits 6 proteins, which resulted in decreasing boar sperm linear motility [2]. Addition of antioxidants to the extender helps to scavenge the excessive ROS to maintain sperm quality [2]. In the present study, we found that addition of cysteine enhanced biosynthesis of GSH, decreased the ROS level, lipid peroxidation and 4-HNE modifications, and thus improved sperm quality during the liquid storage.

Artificial insemination (AI) is commercially applied worldwide on pig farms [36,37]; however, the technique is still not efficient because a large dosage of sperm per sow in estrus is required for getting high reproductive performance (5 to 7×10^9^ sperm for per sow fertilization) [36,38]. The required large number of sperm for successful fertilization is a critical limitation for AI application [36,38]. In addition, liquid storage semen is widely in common use in AI of pig production. And improvement of the post-storage sperm quality is one of the key points for reducing the total sperm numbers per AI. Thus, developing novel boar semen extender will help to improve pig production. In present study, we found that addition of cysteine significantly improved sperm quality via enhancing the GSH biosynthesis during the liquid storage. The novel insight that boar sperm could biosynthesise GSH might provide a new strategy for developing the extender and improving the AI technique in pigs.

## CONCLUSION

**Figure 8:**
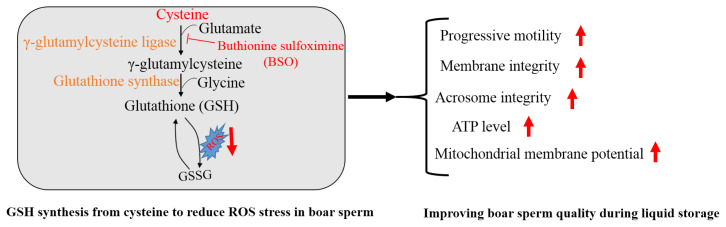
Mechanisms of cysteine involved in regulating boar sperm quality. Boar sperm could utilize the cysteine to generate GSH for reducing the ROS stress, thus improving sperm quality. BSO, buthionine sulfoximine.

Addition of cysteine improved boar sperm progressive motility, mitochondrial membrane potential, ATP production, membrane integrity and GSH content, while it lowered the ROS level, lipid peroxidation and 4-HNE modifications in boar sperm during the liquid storage. Moreover, boar sperm expressed the GCLC and GSS enzymes that were involved in GSH biosynthesis. In the menadione-induced oxidative stress model, addition of cysteine enhanced GSH biosynthesis and reduced the ROS stress, while BSO, which is an inhibitor of GSH generation, counteracted the positive effects. Therefore, cysteine protects boar sperm against ROS-induced damages during liquid storage via enhancing GSH biosynthesis (Figure 8).

## Figures and Tables

**Table 1:** Effects of different concentrations of cysteine on sperm total motility during the liquid storage

L-cysteine (mM)	Day 1	Day 5	Day 9	Day 13
0	90.52±0.90	87.46±1.64	85.17±2.02	85.36±0.84^b^
0.625	92.05±1.04	89.12±1.31	88.10±1.45	87.28±1.14^ab^
1.25	91.57±0.94	90.94±0.57	88.79±1.31	88.92±1.11^a^
2.5	91.55±0.96	90.25±1.02	89.06±1.09	87.93±0.89^ab^
5	91.44±0.93	90.01±0.81	89.31±0.77	88.26±0.91^ab^

Results are expressed as mean±standard deviation.

a,b Different lowercase letters indicate significant differences (p<0.05).

**Table 2:** Effects of different concentrations of cysteine on sperm progressive motility during the liquid storage

L-cysteine (mM)	Day 1	Day 5	Day 9	Day 13
0	81.19±1.04	77.31±0.98^c^	74.20±1.78^b^	71.60±0.99^b^
0.625	82.08±0.93	78.88±0.81^bc^	76.62±1.26^ab^	73.85±0.84^ab^
1.25	82.76±0.85	81.51±0.46^a^	79.04±0.91^a^	75.78±1.10^a^
2.5	82.82±0.97	80.23±0.58^ab^	78.01±1.22^ab^	74.50±0.94^a^
5	82.79±0.90	80.51±0.45^ab^	78.05±0.96^ab^	74.77±0.79^a^

Results are expressed as mean±standard deviation.

a–c Different lowercase letters indicate significant differences (p<0.05).

**Table 3:** Effects of different concentrations of cysteine on sperm straight-line velocity (VSL, μm/s) during the liquid storage

L-cysteine (mM)	Day 1	Day 5	Day 9	Day 13
0	47.26±4.64	43.46±4.21^b^	40.49±3.24^b^	38.15±2.37^b^
0.625	46.75±3.87	47.11±2.14^ab^	46.24±3.94^ab^	43.59±2.93^ab^
1.25	49.31±3.03	50.54±1.63^a^	48.41±1.28^a^	46.92±0.77^a^
2.5	47.08±2.68	47.03±2.30^ab^	47.21±3.46^ab^	44.92±2.85^a^
5	48.67±2.52	47.08±1.58^ab^	48.35±3.12^ab^	45.17±3.67^ab^

Results are expressed as mean±standard deviation.

a,b Different lowercase letters indicate significant differences (p<0.05).

**Table 4:** Effects of different concentrations of cysteine on sperm curvilinear velocity (VCL, μm/s) during the liquid storage

L-cysteine (mM)	Day 1	Day 5	Day 9	Day 13
0	105.08±4.15	99.67±2.99^b^	97.02±3.47^b^	94.95±3.29^b^
0.625	104.97±3.50	103.92±5.11^ab^	101.81±5.12^ab^	97.59±3.70^b^
1.25	107.61±1.67	106.63±0.51^a^	108.21±1.17^a^	105.70±1.52^a^
2.5	104.18±2.55	103.79±2.63^ab^	101.72±2.73^b^	100.37±3.36^b^
5	105.19±4.60	102.87±3.20^ab^	100.99±4.91^b^	98.71±3.16^b^

Results are expressed as mean±standard deviation.

a,b Different lowercase letters indicate significant differences (p<0.05).

**Table 5:** Effects of different concentrations of cysteine on sperm beat-cross frequency (BCF, Hz) during the liquid storage

L-cysteine (mM)	Day 1	Day 5	Day 9	Day 13
0	8.78±0.19	7.67±0.53	7.14±0.20^b^	7.09±0.24^c^
0.625	8.79±0.37	8.02±0.48	7.52±0.48^ab^	7.30±0.22^bc^
1.25	8.59±0.42	8.37±0.28	8.01±0.28^a^	8.06±0.22^a^
2.5	9.05±0.28	8.36±0.27	8.14±0.15^a^	7.76±0.16^ab^
5	9.13±0.26	8.44±0.26	8.24±0.17^a^	7.76±0.21^ab^

Results are expressed as mean±standard deviation.

a–c Different lowercase letters indicate significant differences (p<0.05).
